# Occupational Exposure to Solar Radiation at Different Latitudes and Pterygium: A Systematic Review of the Last 10 Years of Scientific Literature

**DOI:** 10.3390/ijerph15010037

**Published:** 2017-12-26

**Authors:** Alberto Modenese, Fabriziomaria Gobba

**Affiliations:** Department of Biomedical, Metabolical and Neurosciences, University of Modena & Reggio Emilia, 41124 Modena, Italy; f.gobba@unimore.it

**Keywords:** pterygium, solar ultraviolet radiation, occupational eye exposure, outdoor work, worker health prevention

## Abstract

Pterygium is a chronic eye disease: among its recognized risk factors there is long-term exposure to ultraviolet (UV) radiation. The Sun is the main source of UV exposure: according to the World Health Organization, the Population Attributable Fraction of pterygium due to solar radiation (SR) is 42–74%. Outdoor work can deeply influence the eye exposure to solar UV rays, but, despite this, pterygium is currently not adequately considered as a possible occupational disease in this working category, at least in Europe. For this reason, we performed a systematic review of the scientific literature published in the last ten years (2008–2017) considering the role of outdoor work as a risk factor for pterygium, in order to give new support for the prevention of this UV related disease in workers. We identified 29 relevant papers. Our results show that pterygium prevalence highly increased with latitude and mean annual UV index, and outdoor work is one of the most relevant risk factors, as well as age and male sex, both in high risk and in moderate risk World areas considering the environmental UV levels. Accordingly, pterygium occurring in outdoor workers should be considered an occupational disease. Moreover, our findings clearly support the need of further research on more effective prevention of the occupational risk related to long-term solar radiation exposure of the eye.

## 1. Introduction

Pterygium is a “wing-shaped” abnormal growth of the conjunctiva onto the cornea, that can cause ocular irritation, a relevant cosmetic effect and, in the late stage of the corneal tissue’s invasion, visual impairment [1]. The only available treatment is the surgical removal of the lesion, but unfortunately pterygium may often relapse. Actually there isn’t an effective medical therapy for this disease, although certain drugs, mainly lubricants, vasoconstrictors and topical corticosteroids, are used to control subjective symptomatology [2]. 

The lesion occurs often bilaterally, although with asymmetric development [3]. Even in cases of mono-laterality, pterygium is frequently associated to an initial degeneration of the contralateral conjunctival epithelium, without involvement of the cornea: the pinguecula [4]. The prevalence of this disease is highly variable, ranging from 1.1 to 40% in different groups [5,6] investigated in various places of the world; taken as a whole data suggest an inverse correlation with the latitude. Furthermore, the prevalence increases with age and it is higher in male subjects [7].

Knowledge on the pathogenesis of this disease is still incomplete, but data indicate a role played by genetic, infectious, immunological and environmental factors, including long-term exposure of the ocular surface to solar UV radiation (UVR) [1,4,7,8,9,10]. This latter hypothesis is supported by the greatest risk of pterygium observed in populations living in tropical regions [6,7,8,9,10]. According to WHO (World Health Organization), the Population Attributable Fraction (PAF) of pterygium due to solar radiation (SR) exposure is 42–74% [7].

A relevant and well known factor influencing long-term eye exposure to solar UV is outdoor work [6,7,8,9,10,11]: accordingly in outdoor workers (OW) a higher risk of pterygium should be expected. The first description of pterygium in an OW is in a case report published in 1876 on a 65 years old male farm-bailiff of with bilateral severe lesions [12]. A non-systematic review on the relation between eye UV exposure and pterygium was published in 2009, in a case report of a bilateral pterygium in an Israeli tennis instructor [13]. The conclusion of the Authors was that pterygium may be considered an occupational disease in outdoor workers. At our knowledge, no other reviews were subsequently published on this topic. According to World Health Organization, an “occupational disease” is any disease contracted primarily as a result of an exposure to risk factors arising from work activity. Pterygium is among the eye effects included in the ICNIRP/ILO/WHO publication “Protecting Workers from Ultraviolet Radiation” [8], in the “Non-binding guide to good practice for implementing Directive 2006/25/EC-Artificial Optical Radiation”, published in 2010 by the European Commission [9], and also the 2012 document “Health Effects of Artificial Light” of the Scientific Committee on Emerging and Newly Identified Health Risks (SCENIHR), stated that outdoor work is a “recognized factor for pterygium development” [10]. A detailed description on the general criteria for identification and recognition of occupational diseases is reported in the International Labour Organization (ILO) publication OSH Series 74 “List of Occupational Diseases” (2010) [14]: a specific discussion on this topic is not the aim of this review, but on our opinion an update on new epidemiological data on the relation between occupational exposure to solar UV and pterygium published in the scientific literature after the review of Maharshak and Avisar [13] is needed, to further study its association with outdoor work. 

Considering these premises, we performed a systematic review of the scientific papers published during the last ten years (2008–2017) specifically considering the role of outdoor work as a risk factor for pterygium.

## 2. Materials and Methods 

According to the “Preferred Reporting Items for Systematic Reviews and MetaAnalyses” [15], Medline (through PubMed) and Scopus databases were searched with the following search string: [“pterygium” AND (work* OR job* OR occupation*) AND (“outdoor” OR “solar radiation” OR sunlight OR UV)]. Only original research articles with an available English abstract published in peer-reviewed journals in the period 1 January 2008–31 October 2017 were collected, while reviews, case reports, comments or letters were excluded. Two main types of studies were considered eligible for inclusion:

- studies on the presence of pterygium in groups of outdoor workers, or in groups of the general population investigated also for the occupational solar radiation exposure;
- studies in groups of patients with pterygium investigated for the occupational solar radiation exposure or, at least, classified as outdoor or indoor workers, based on their job title.

Data extraction was performed by one reviewer and checked by another. The abstracts of all the studies collected were analyzed: for all of the studies meeting the inclusion criteria, full papers were retrieved. Then, references of the selected papers were also checked to find other relevant studies.

The UV risk of the workers investigated in the studies was further estimated based on the latitude of the workplaces (i.e., the places where the studies were performed): for each study, we calculated, and rounded to the nearest unit, the mean annual UV index (UVI) related to that latitude. This calculation was based on the monthly UVI data reported on the World Health Organization INTERSUN programme webpage [16] for a range of cities in different countries (according to WHO, the reported UVI values were measured on the 21st of each month and the maximal values were taken). Then, according to the mean UVI, the workplaces were ranked in 5 Risk Categories: “low” (UVI between 0 and 2), “moderate” (UVI 3–5), “high” (UVI 6–7), “very high” (UV 8–10) and “extremely high” (UCVI > 11). Finally, we compared the mean pterygium prevalence and the mean Odds Ratios reported in the studies conducted in high/very high/extremely risk areas versus research performed in moderate risk areas.

## 3. Results

**Scheme 1:**
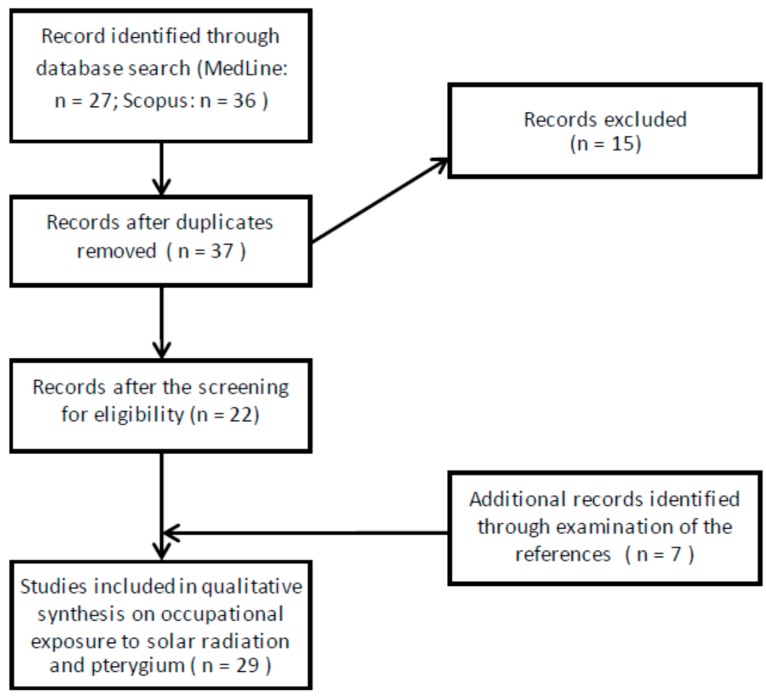
Flow Diagram describing the stages of the search process of the systematic literature review on occupational exposure to solar UV radiation and pterygium.

The electronic search of literature databases at 31 October 2017 resulted in 63 hits (PubMed = 27, Scopus = 36), 37 after the removal of the duplicates. After the screening of the abstracts for the eligibility, we identified 22 relevant articles. At a hand search of the references of the articles, 7 additional articles were identified, giving a total of 29 papers included in this systematic review of scientific literature of the last 10 years (2008–2017) on occupational exposure to solar UVR and pterygium (Scheme 1).

The results of the classification of the studies according to UV risk estimated from mean UV Index are shown in Table 1: 12 studies were performed in areas at “extremely high” risk, 7 in areas with a “very high” risk; 4 in high risk areas and 6 studies in places with a “moderate” risk, while no published studies were performed in “low risk” areas (mean annual UVI < 3).

Here below we present the main findings of these studies; the results are summarized in the Table 2, Table 3, Table 4 and Table 5.

### 3.1. Areas at Moderate UV Risk

Six studies on occupational exposure to solar radiation and pterygium have been found in areas at moderate risk considering the mean annual UV index in last ten years: 1 from Europe (Spain) [17], 2 from China [18,19] and 3 from South Korea [20,21,22] (Table 2).

Viso et al. (2011) [17] evaluated pterygium prevalence in North-West Spain in 619 subjects >40 years, of which 37.0% were males. Pterygium prevalence was 5.9%, increasing significantly with age (*p* < 0.005), and was higher in females (6.5% vs. 4.8% in males). Pterygium was significantly associated with outdoor activity, with an OR of 2.28 (95% CI: 1.04–4.98). Another factor positively associated with the disease was fluorescein staining (OR = 2.64; 95% CI: 1.08–6.46).

In China (Beijing area) the longitudinal study of Zhao et al. (2013) [18] found a 10-Years pterygium incidence in 2628 subjects (mean age 40 years at the baseline, pterygium prevalence = 2.5% of 2695 subjects) of 4.9%. Farmers were more frequently represented in pterygium group and the OR at the univariate analysis was 3.67 (95% CI: 2.5–5.4) versus non farmers, but the association was not significant at multivariate analysis (*p* = 0.47), where only the rural region of habitation (*p* < 0.001) and a lower fasting blood glucose concentration (*p* = 0.009) were significant. In the rural area of North China Li et al. [19] investigated 8445 subjects, aged >18 years: the overall pterygium prevalence was 2.5% (1.2% bilateral) (95% confidence interval (CI): 1.0–1.4%). According to multivariable analysis, pterygium presence was significantly associated with outdoor work (OR: 1.8; 95% CI: 1.2–2.6), but also with age >70 years (OR: 29.0; 95% CI: 13.6–61.6) and male sex (OR: 1.9; 95% CI: 1.4–2.6), while no association was found for smoke and alcohol use history.

Three large national South-Korean studies have been included in the review [20,21,22]. Lim et al. [20] collected data from a large sample of 13,204 individuals (age 40 years or older), 821 diagnosed with pterygium and 12,383 controls. The frequency of outdoor workers was higher in cases than in controls (38.2% vs. 21.9%, *p* < 0.001), but in a multivariate analysis only the reporting of >5 h per day outdoor was significantly associated (OR: 1.47; 95% CI: 1.11–1.94). Other risk factors identified were age (OR: 1.05; 95% CI: 1.05–1.06) and male gender (OR: 1.28; 95% CI: 1.01–1.61) and lower level of education (OR: 3.98; 95% CI: 2.24–7.06), while urban residency was negatively associated (OR: 0.56; 95% CI: 0.45–0.70). In another South Korean population based study, Nam et al. [21] investigated 16,234 adults with an overall pterygium prevalence of 7.1%. Outdoor workers were more represented in the pterygium group compared to controls, both in males (2.6% vs. 1%, *p* < 0.001) and in females (2.9% vs. 0.7%, *p* < 0.001). Also the reporting of ≥5 h per day of sun exposure was positively associated: 2.6% vs. 0.9% in males (*p* < 0.001) and 3% vs. 0.7% in females (*p* < 0.001). This study was primarily aimed to investigate the association between pterygium and obesity, finding a significant association in obese women: OR = 1.35 (95% CI: 1.02–1.77). Finally, in the 2 years prospective study of Lee et al. [22] on 9839 adults aged 19–74 years, pterygium incidence was of 6%. Pterygium frequency was almost 3 times higher in OW than in indoor workers (OR: 3.1; 95% CI: 1.9–4.8 at a multiple logistic regression analysis). Low educational status and longer working hours were also significantly associated.

### 3.2. Areas at High UV Risk

Four studies on occupational exposure to solar radiation and pterygium have been found in areas at high risk considering the mean annual UV index in last ten years (Table 3): 2 from Iran [23,24], 1 from Japan [25] and 1 from U.S. [26]. 

Rezvan et al. [23] reported on 5190 people from the urban population of Shahroud (Iran), who underwent an ophthalmological examination: pterygium prevalence was 9.4% (95% CI: 8.6–10.3), 2.9% (95% CI: 2.4–3.3) bilateral. A significant difference in pterygium prevalence was found between OWs and indoor workers (14.7% vs. 8.4%); furthermore, the prevalence of pterygium in OWs spending half time outdoor and half time indoor was 9.3%, compared to 14.7% in full time OW: the difference is significant (*p* < 0.001), supporting an exposure-effect relation. Pterygium was found to be significantly associated also with male sex (11.4% in males vs. 8.0%), and with age only in females. The other risk factor observed was a low educational level: pterygium prevalence was higher in illiterates than in subjects with a college education (16.5% vs. 5.4%, *p* < 0.001). In the other Iranian study Malekifar et al. [24] investigated 210 pterygium or pinguecula cases and an equal number of controls from the dry and high altitude province of Ilam. Outdoor occupation (*p* < 0.001) and also welding experience (*p* < 0.001) were significantly associated with pterygium at the univariate analysis, while in multivariate analysis only family history of pterygium, cigarette smoking, history of baking, age and severe blepharitis were significantly associated (*p* < 0.001, *p* < 0.001, *p* = 0.002, *p* = 0.023 and *p* = 0.002, respectively) and, among OWs, only farmers showed a significant OR of 3.84 (95% CI: 1.01–14.67, *p* = 0.049) for having pterygium.

Tano et al. [25] provided an ocular examination to 2312 subjects in Northern Japan, mean age 64.3 years: pterygium prevalence was 4.4%. OW were more represented in the pterygium group than in controls (29.7% vs. 22.3%), with a slightly higher prevalence of 5.7% (OR = 1.47; 95% CI: 0.9–2.3, not significant) and in particular in those with a bilateral disease (36.1% vs. 22.5%, OR = 1.95; 95% CI: 0.98–3.9, not significant). Only age was found to be significantly associated with pterygium in a multivariate analysis.

In the 2009 study of West et al. [26] a population-based sample of 4774 Hispanic subjects aged >40 years and living in Arizona (US) was collected. The participants were examined by an ophthalmologist and the pterygium prevalence was 24% in men, compared with 11% in women. OWs were more likely to be male, with low economic income and low educational level: both these three factors were significantly associated with the presence of pterygium. Male sex was associated with an OR = 2.67 (95% CI: 2.2–3.2). A low educational level was positively associated with an OR = 2.8 (95% CI: 2.2–3.6) for less than 6 years spent at school in total and also for a period of 6–11 years (OR = 1.8; 95% CI: 1.4–2.2) versus those with a scholarity higher than 11 years in total. Also the mean annual economic income was significant different between pterygium group and controls, adjusted for age and sex (*p* < 0.001). Other factors associated were the history of diabetes (*p* = 0.03), smoke (*p* = 0.02) and of bilateral cataract surgery (*p* = 0.004), while hypertension, alcohol use and any cataract presence were found to be not associated.

### 3.3. Areas at Very High UV Risk

Seven studies on occupational exposure to solar radiation and pterygium have been found in areas at very high risk considering the mean annual UV index in last ten years (Table 4): 1 study from the Norfolk Islands in Australia [27], 5 studies from Asia, of which 2 from South China [28,29], 1 from South Japan [30], 1 from North India [31] and 1 from Taiwan [32], and 1 study from the Hawaii islands in U.S. [33].

The Australian study by Sherwin et al. (2008) [27] was aimed to investigate the association between conjunctival ultraviolet autofluorescence (UVAF), a biomarker of ocular UVR exposure, and pterygium. 641 residents aged ≥15 years of the Norfolk Island in the South Pacific completed a sun exposure questionnaire and underwent autorefraction and ocular examination. The overall pterygium prevalence was 10.9%, higher in males (15% vs. 7.7% in females, *p* = 0.003). Spending more than three-quarters of the day outside was significantly associated with pterygium presence at a multivariate analysis with an OR of 2.22 (95% CI: 1.20–4.09, *p* = 0.011). Males who spent more than the 75% of the day in outdoor activities were more likely to be OW. Other positively associated factors with pterygium were UVAF per 10 mmq (OR 1.16; 95% CI: 1.16–1.28) and a tanning skin phenotype (OR 2.17; 95% CI: 1.20–3.92).

Zhong et al. [28] investigated a large Bai nationality group in south-west China with 2133 subjects >50 years: pterygium was associated with outdoor work with an OR of 1.51 (95% CI: 1.10–1.92). Other factors associated were increasing age (significant OR ranging from 1.47 to 1.79 according to the age group, female sex (OR: 1.42; 95% CI: 1.08–1.88), low education level (OR: 1.26; 95% CI: 1.03–1.56), while BMI, hypertension, diabetes, smoking and alcohol use history were not associated. The same Bai group was investigated after 5 years by Li et al. (2015) [29] to determine the 5-year pterygium incidence, that was of 6.8% (95% CI: 5.2–8.4), significantly higher in women than in men (8.8% vs. 3.8%; *p* = 0.003). Outdoor occupation was the only significant associated variable at multivariate analysis: OR = 2.52 (95% CI: 1.27–4.95).

A Japanese study [30] was conducted in the population aged >40 years on Kumejima Island: 3762 subjects were examined and pterygium prevalence was 30.8% (13.1% bilateral). History of outdoor work was found to be significantly associated with a higher pterygium risk in a logistic regression analysis: OR = 1.82 (95% CI: 1.33 to 2.50). The frequency of OWs was 77.8% in the pterygium group vs. 59.0% in controls (*p* < 0.001). Considering the main occupations, farmers were the 31.8% of the pterygium group vs. the 22.4% of the controls (*p* < 0.001), and fishermen were 4.2% vs. 2.4% (*p* = 0.003). Other positive associations with pterygium were found for older age (OR = 1.02; 95% CI: 1.01–1.03), male sex (OR = 1.33; 95% CI: 1.03–1.63), hyperopic refraction (OR = 1.08; 95% CI: 1.03–1.13), while a negative association was found with the values of the intraocular pressure (OR = 0.96; 95% CI: 0.94–0.98).

In a nine months prospective study conducted in Northern India [31] on 200 patients treated for pterygium, Authors observed that the frequency of the disease increased with the numbers of hours usually worked outdoor (6–8 h on average), not further quantifying this suggested association. 

The Taiwanese study of Chen et al. [32] conducted in the rural area of the Chiayi County (South Taiwan) considered 2197 subjects aged >40 years: pterygium prevalence was 25.2%, increasing with age (30.1% in subjects 60–60 years, *p* < 0.0001). OWs were found to be at a higher risk for having pterygium, with an OR of 1.47 (95% CI: 1.17–1.86). Other risk factors were male sex (OR = 1.31; 95% CI: 1.08–1.60), smoking history (OR = 1.36; 95% CI: 1.02–1.83) and living in a seaside area (OR = 1.65; 95% CI: 1.35–2.03).

Finally, a U.S. study [33] was conducted in the Oahu Island, Hawaii, in a group of 169 subjects aged 18–80 years: 52.1% were non-surfers, 24.3% occasional surfers, 8.9% recreational surfers and 14.8% professional surfers, based on their lifetime surfing hours. The overall pterygium prevalence was 11.9% (5.3% bilateral). Also the history of outdoor occupation, other than professional surfing, was significantly associated with pterygium (*p* = 0.04), as well as lifetime surfing hours (*p* < 0.0001). Other associated factors were Hawaiian residence (*p* = 0.009) and Hawaiian/Pacific Islander ethnicity (*p* = 0.002).

### 3.4. Areas at Extremely High UV Risk

Twelve studies were conducted in world areas at extremely high risk according to the mean annual UVI: three in Africa (Ghana [34], Nigeria [35] and Ethiopia [36]), two in Central/South America (Barbados [37] and Brazil [38]) and seven in Asia (four from South India [39,40,41,42], one from Thailand [43] and two from Singapore [44,45]) (Table 5).

In Africa, Essuman et al. [34] performed a prospective study of 60 consecutive patients from Ghana, aged 17–75 years, treated for primary pterygium with a surgical excision and then followed after the operation for 30 months. 58% of the patients were females. The overall frequency of pterygium recurrence was 37%. No significant association was found between pterygium recurrence and outdoor occupation. The study by Achigbu and Ezepue [35] investigated a group of 615 commercial motorcyclists, mean age 38.1 years, in South-Eastern Nigeria. The prevalence of pterygium was 19.3%. Considering the severity of the disease, the majority of these OW had an early stage lesion (52%), while the 46% of the sample had a medium stage and the remaining 2% had the most severe lesions. Pterygium was significantly associated with the length of employment as commercial riders (*p* = 0.009). Furthermore, the authors reported a lack of protective effect of regular use of sunglasses during work (*p* = 0.188). Anbesse et al. [36] studied pterygium prevalence in 390 subjects, mean age 38.7 years, living in Northwest Ethiopia, finding that the 38.7% of the participants were affected by the disease. OWs resulted at a significantly higher risk for pterygium (OR = 3.8; 95% CI: 2.18–6.46), and the regular use of sunglasses and hat was a protective factor (OR = 0.40; 95% CI: 0.2–0.78). Other associated factors were age classes (OR = 2.20; 95% CI: 1.22–3.39 for the age class 41–60 years, OR = 7.97; 95% CI: 2.74–23.17 for the age class 61–86 years), male sex (OR = 2.20; 95% CI: 1.28–3.82), use of traditional eye medications (OR = 2.55; 95% CI: 1.04–5.9) and inheritance for pterygium (OR = 6.68; 95% CI: 2.53–17.6).

Considering Central/South American studies, Nemesure et al. [37] conducted a longitudinal study in the Barbados Islands, evaluating the nine-year incidence of pterygium in a sample of 1888 participants, aged 40–84 years at the baseline. After a 9-year follow up the development of pterygium was observed in the 11.6% of the subjects, for an average of 1.3% incidence per year, with no significant association with increasing age. OW were found at a higher risk for pterygium occurrence at a multivariate analysis (OR = 1.51: 95% CI: 1.05–2.16). Darker skin pigmentation and glasses/contact lenses use were reported to be significant protective factors (OR = 0.67; 95% CI: 0.46–0.97 and OR = 0.58; 95% CI: 0.42–0.81, respectively). Another South American study was conducted by Coutts et al. in Amazonia region of Brazil in 2011 [38]. The study included 225 subjects aged >20 years living in three different places: Manaus city (*n* = 89), on the riverside (*n* = 116) and inside the rainforest (*n* = 20). Pterygium prevalence was 52%, lower in Manaus city, higher close to the river (62.5%) and much higher inside the rainforest (75%). Overall, the 46% of the subjects had a bilateral lesion. Among the rainforest inhabitants, the percentage of OW reached the 70%, and the 67% of people living on the riverside, while OWs were only the 31% of the Manaus participants. In this group, subjects who worked outdoor showed an increase in the severity of pterygium: OWs represented the 57% of the subjects with grade 2 pteygium in this group, while they were the 93.3% of patients with a grade 3 lesion (*p* = 0.0001) and the 100% of the subjects with the most severe lesions (grade 4, *p* = 0.0004).

Among the seven Asian studies, four were from South India [38,39,40,41]: in these studies outdoor workers were found to be at risk for pterygium development with a significant OR = 1.8 (95% CI: 1.5–2.2, *p* < 0.001) in the large population study conducted by Marmamula et al. [40] in 10,293 subjects, while in the study by Asokan et al. [38] considering the general population of an urban and a rural district (7774 subjects) the type of work was not associated with pterygium: only rural residence and higher lifetime UV exposure were significantly associated with the presence of pterygium (*p* < 0.0001). In these two studies also other pterygium risk factors were considered: Asokan et al. found a pterygium prevalence of 9.5%, higher in subjects reporting a non-use of glasses (OR: 1.41; 95% CI: 1.12–1.79), while smoking, use of alcohol, diabetes and hypertension were not associated. Marmamula et al. found that the prevalence of pterygium (mean 11.7%) increased with age and for those who lived in rural areas (OR = 1.8, 95% CI: 10.9–12.6), while higher education level had a protective effect (OR = 0.6; 95% CI: 0.5–0.7; *p* < 0.001). The prospective study of Salagar et al. (2013) [41] on South-Indian patients treated for pterygium showed that the 80% of 100 patients followed for 1 year were OWs, with a higher frequency of complicated pterygia (X_2_ = 7.7, *p* < 0.001). Another Indian study was conducted in 331 salt workers with a mean age of 41.9 years by Cherian et al. [39]: these OWs were exposed to sunlight with high reflections and the pterygium prevalence was 13%, higher in males.

A study conducted in Thailand (Artornsombudh et al. [42]) was aimed to investigate the prevalence of conjunctival neoplasia in patients diagnosed for pterygium: 482 subjects, mean age 56.5 years, were studied and the prevalence of conjunctival epithelial neoplasia was approximately 1.8%. Among the patients, the 53.7% were farmers, 18.7% laborers, 4.6% merchants, 1% truck drivers and 22% pure indoor workers.

In Singapore the prevalence of pterygium in an urban Malay population of 3280 subjects aged 40–79 years (Cajucom et al. [43]) was 12.3% (4.9% bilateral). The severity of pterygium was graded on 3 levels, and the history of outdoor occupation was associated only with severe pterygium (Grade 3) at multivariate analysis (OR = 2.2; 95% CI: 1.1–4.5, *p* = 0.03). Pterygium of any grade was independently associated with increasing age (OR = 1.3; 95% CI: 1.1–1.4), male sex (OR = 1.9; 95% CI: 1.5–2.6) and high systolic blood pressure (OR = 1.6; 95% CI: 1.2–2.1). The other study performed in Singapore by Ang et al. (2012) [44] considered three different ethnic groups Malays, Indians and Chinese persons >40 years of age for a total of 8906 participants who underwent an ocular examination. The overall pterygium prevalence was 10.1%, while severe pterygium was diagnosed in 1.6% of the subjects. The prevalence was higher in Malays (15.5%) than in Chinese (7.0%; *p* < 0.001) or Indians (7.0%; *p* < 0.001). Only severe pterygium was associated with outdoor occupation (OR = 2.1; 95% CI: 1.1–4.0; *p* = 0.02), and the ethnic group did not affected this association. Other pterygium associated factors in a multivariate analysis were increasing age (*p* < 0.001), male sex (*p* < 0.001) and low education level (*p* < 0.001).

### 3.5. Mean Pterygium Prevalence and Odds Ratios for Likelihood of Outdoor Workers to Have Pterygium According to the UV Risk Areas

In Table 6 the mean pterygium prevalence found in the studies reviewed are summarized according to the UV risk areas and an average weighted prevalence considered the number of subjects studied have been calculated. Our results show a mean crude pterygium prevalence of 19.3% in high/very high/extremely high areas vs. 4.8% in moderate risk areas. The difference is reduced to 14.9 vs. 5.5 considering pterygium prevalence weighted for the number of subjects investigated in each study (Table 6).

In Table 7 the mean Odds Ratios for outdoor workers to have pterygium are shown: considering high/very high/extremely high risk areas we calculated a mean OR of 2.2, based on 12 studies, and the same average OR also resulted from five studies of the moderate risk area (Table 7).

## 4. Discussion

Considering long-term solar radiation exposure, taken as a whole data presented in our review further support the role of solar UV radiation in pterygium induction, as shown by the 3–4 times higher pterygium prevalence in high/very high/extremely risk areas for the mean annual UVI versus moderate risk areas (Table 6).

Specifically considering occupational solar UVR exposure, our results show that pterygium is a frequently occurring disease in outdoor workers occupationally exposed to Solar Radiation for many hours during the day for many years during their working life: significant ORs in reviewed studies were found, ranging from 3.8 in Iran and Ethiopia [24,36] to 1.4 in Taiwan [32], and the mean ORs in different UV risk areas do not differ (Table 7). Accordingly, outdoor workers had increased odds, from 50 to 380%, of pterygium, and these odds did not vary significantly with latitude: occupational exposure to solar UV radiation is one of the most relevant factors associated to pterygium presence, both in case of high-extremely high levels of environmental UV and also in case of a moderate UV index.

Occupational exposure was found to be positively associated also with the severity of the disease [38,42,44,45]. Furthermore in one study pterygium was specifically associated with length of employment [35]. Considering protective factors, the use of sunglasses and hat, but also simple eyeglasses, reported a negative association with pterygium in OWs [36,37,39], even if one study found a lack of protective effect of sunglasses use in a sample of professional motorcyclists in Africa [35]. 

Another interesting point is the high number of scientific studies published in a quite short period (10 years) on the topic of occupational exposure to solar radiation and pterygium: we found a total of 29 studies, conducted in different regions of the World. The areas with the lowest number of studies were Australia and Europe, both with only one study. Considering Australia, it has to be noted that in 80s, 90s and early 2000s several studies have been published by Australian researchers on the topic of chronic UV related eye effects, including pterygium [1,2,6,46,47,48,49,50]. On the contrary, considering Europe, very few studies on this topic have been conducted also prior to 2008: this may indicate a lack of attention in Europe to the problem of UV-induced pterygium in outdoor workers, despite the WHO estimates for pterygium an annual loss of 360 Disability Adjusted Life Years (DALYs), in particular in Mediterranean Europe [7]. Furthermore, the European study we found, conducted in the North-West of the Spain [17], showed a relevant pterygium prevalence (5.9%) and significant positive OR for OWs (2.3): this indicates a substantial underestimation of this problem in Europe, and the need of a further consideration in European outdoor workers.

In agreement with previous results our review confirms some other pterygium risk factors, as aging and male gender, even if in particular categories, as in example in some Chinese rural populations where women spend the majority of their time outdoor, pterygium prevalence was higher in females [28,29].

### Limitations of the Systematic Review

The quality of the analysis performed in the studies was non-uniform and quite often modest, especially if we consider the methods adopted to evaluate occupational exposure to solar UV. In order to give a comprehensive panorama of recent scientific literature published on the topic of pterygium in outdoor workers we decided not to exclude any of the studies identified, but we have to observe that a more adequate exposure assessment is necessary to provide epidemiological evidences for pterygium as a possible occupational disease related to UV exposure at work in outdoor and/or indoor settings. Moreover, the different study-designs and methodologies applied precluded any possibility of a meta-analysis. Among the major source of bias of the reviewed studies we can cite here:

- Study Design
  No randomized control trials have been found among the studies reviewed. The majority of the studies are prevalence studies with a cross-sectional design, that, apart from being not an appropriate design to assess causality, it is also prone to selection and recall bias.
- Exposure Evaluation
  The majority of the studies provides an exposure evaluation based on a definition of OWs only according to the job title (e.g., farmer) or on a subjective retrospective evaluation of the time spent outside during work, with no quantitative measures of solar UVR as well as adjustments for non-occupational exposures, determining a clear risk of recall bias. The bias can be related to the non-blind attribution of pterygium patients and, if investigated, of controls, to the exposed/not exposed working categories: no study has a blind categorization method, enabling a bias in both directions, depending on the conviction of the patient or scientist. It has to be also noted that job titles are not a reliable estimate of the exposure: solar UV may largely differ within the same working groups according to the specific working tasks performed, the working environment and the use of protections, etc. [8]. On the other hand, the studies providing a subjective exposure evaluation are prone to recall bias.
- Outcome Definition
  In the majority of the studies the criteria for the diagnosis of the disease are not adequately explained, and it is not possible to be sure, as in example, if the outcome is really a pterygium (ICD-10 code: H11.0) or a pseudo-pterygium (H11.8), and also the classification of the severity of the lesions, when considered, is based on different methods and parameters.

Considering as a whole these limiting conditions, it is also not possible to recognize a unique direction of the bias, so that the association between outdoor work and pterygium may be either overestimated or underestimated. Further limitations may regard other occupational factors possibly relevant for pterygium development: as stated in the Methods section, we did not consider in this review studies investigating occupational artificial UV exposure, as in example in welding activities [5,8,51,52]. Furthermore, there are possibly other work-related factors associated with chronic corneal and conjunctival irritation that may be considered in pterygium etiology [2,3,4,53,54], including repeated occupational eye injuries, quite frequent in outdoor workers [55,56], e.g., in case of eye trauma with penetration of dusts in the eye [57], especially considering that outdoor work may be performed in a windy/sandy environment [47,58]: these factors were not adequately considered in the reviewed studies, and their role may deserve further investigations.

## 5. Conclusions

In our systematic review of recent scientific literature published on the topic of occupational exposure to solar UVR and pterygium occurrence we found that the prevalence of this disease highly increased at low latitudes with a high mean annual UVI, in particular in outdoor workers, but occupational exposure to solar UV radiation is one of the most relevant risk factors for pterygium also at quite high latitudes with low pterygium prevalence. Due to the limitations of the studies reviewed, related in particular to their design, exposure evaluation and outcome definition, the results of the studies presented in this review can only be considered in favor of the hypothesis of a possible association between occupational solar UV exposure and pterygium, according to the epidemiological evidence of an increased prevalence of the disease in outdoor workers, confirming the statements of institutions/scientific organization as WHO, ILO, ICNIRP, SCENIHR and the European Commission. New research with more adequate occupational exposure assessments is needed to further support the possible inclusion of pterygium among occupational diseases. Furthermore, our findings clearly support the need of further research also on more effective prevention of the occupational risk related to long term solar radiation exposure of the eye, taking into account:

- adequate estimates of cumulative eye exposure to UV, resulting from both occupational and recreational exposure;
- detailed evaluation of the UV exposure, possibly integrating subjective and objective data;
- the role of other occupational factors possibly relevant for the induction and the evolution of pterygium;
- the use of eye protections, in particular consideration of the presence of UV filters and the shape of the glasses, and of brimmed hats/simple caps;
- other relevant information on UV exposure history, e.g., photokeratitis/erythema/sunburns episodes.

Studies with these characteristics should enable a better comprehension of the relationship between outdoor work and pterygium, for the development of a more effective prevention.

## Figures and Tables

**Table 1:** Classification of the reviewed studies on pterygium and outdoor work classified according to the risk category derived for the mean annual UV index; 29 studies included.

Study	Place	Latitude	Risk Classification of Mean Annual UVI
Viso et al. 2011 [17]	O Salne’s (Spain)	42° N	
Zhao et al. 2013 [18]	Beijing (China)	39° N	MODERATE
Li et al. 2014 [19]	Heilongjiang (China)	45° N	
Lim et al. 2015 [20]	South Korea *	37° N **	
Nam et al. 2016 [21]	South Korea *	37° N **	
Lee et al. 2017 [22]	South Korea *	37° N **	
Rezvan et al. 2012 [23]	Shahrud (iran)	36° N	HIGH
Malekifar et al. 2017 [24]	Ilam (Iran)	33° N	
Tano et al. 2013 [25]	Tadami, Minamiaizu (Japan)	37° N	
West et al. 2009 [26]	Arizona (U.S.)	33° N	
Sherwin et al. 2008 [27]	Norfolk Island (Australia)	29° S	VERY HIGH
Zhong et al. 2012 [28]	Yunnan Province (China)	25° N	
Li et al. 2015 [29]	Yunnan Province (China)	25° N	
Shiroma et al. 2009 [30]	Kumejima (Japan)	26° N	
Veena et al. 2013 [31]	Jaipur (India)	26° N	
Chen et al. 2013 [32]	Chiayi County (Taiwan)	23° N	
Lin et al. 2016 [33]	Oahu island, Hawaii (U.S.)	21° N	
Essuman et al. 2014 [34]	Accra (Ghana)	5° N	EXTREMELY HIGH
Achigbu & Ezepue, 2014 [35]	Enugu (Nigeria)	5–7° N	
Anbesse et al. 2017 [36]	Gondar (Ethiopia)	12° N	
Nemesure et al. 2008 [37]	Barbados	13° N	
Coutts et al. 2011 [38]	Manaus (Brazil)	3° S	
Asokan et al. 2012 [39]	Chennai, Tamil Nadu (India)	13° N	
Cherian et al. 2013 [40]	Marakkanam (India)	12° N	
Marmamula et al. 2013 [41]	Hyderabad, Adilabad, West Godavari, Mahbubnagar (India)	16–19° N	
Salagar et al. 2013 [42]	Gulbarga (India)	17° N	
Artornsombudh et al. 2013 [43]	Bangkok (Thailand)	13° N	
Cajucom et al. 2010 [44]	Singapore	1° N	
Ang et al. 2012 [45]	Singapore	1° N	

* National Study; ** Seoul latitude.

**Table 2:** Synthesis of the studies on occupational solar UV radiation exposure and pterygium included in the systematic review of scientific literature published in the period 2008–2017 performed in World areas at moderate risk considering the mean annual UV index (UVI).

Study	Study Design	Place/Latitude	Sample Size (*n*)	Results of Association between Pterygium and Outdoor Work	Other Factors Associated with Pterygium
Viso et al. 2011 [17]	Cross-sectional	North Spain/42° N	619	OR = 2.3 (95% CI: 1.04–4.98)	- Age: (*p* < 0.005) - Female sex : prev 6.5% vs. 4.8% in males (not significant) - Fluorescein staining: OR = 2.6 (95% CI: 1.1–6.5)
Zhao et al. 2013 [18]	Longitudinal	North China/39° N	2628	OR = 3.67 (95% CI: 2.5–5.4) for farmers vs. non-farmers at univariate analysis, not significant (*p* = 0.47) at multivariate	- Living in a rural region: *p* < 0.001 - Lower fasting blood glucose concentration: *p* = 0.009
Li et al. 2014 [19]	Cross-sectional	North China/45° N	8445	OR = 1.8 (95% CI: 1.2–2.6)	- Age > 70 years: OR = 29.0 (95% CI 13.6–61.6) - Male sex: OR = 1.9 (95% CI: 1.4–2.6) - Smoke and alcohol: no association
Lim et al. 2015 [20]	Cross-Sectional Case-control	South Korea/37° N *	13,204	>5 h/day as OWs: OR = 1.47 (95% CI: 1.11–1.94) OWs in pter group = 38.2% vs. 21.9% in controls (*p* < 0.001)	- Age: OR = 1.05 (95% CI: 1.05–1.06) - Male sex: OR = 1.28 (95% CI: 1.01–1.61) - Low education: OR = 3.98 (95% CI: 2.24–7.06) - Urban residency: OR = 0.56 (95% CI: 0.45–0.70)
Nam et al. 2016 [21]	Cross-Sectional Case-control	South Korea/37° N *	16,234	OWs more represented in pter group vs. controls: 2.6% vs. 1%, *p* < 0.001, and in males vs. females: 2.9% vs. 0.7%, *p* < 0.001. ≥5 h/day outdoor: prev = 2.6% vs. 0.9% in males (*p* < 0.001); 3% vs. 0.7% in females (*p* < 0.001)	Obesity in women: OR = 1.35 (95% CI: 1.02–1.8)
Lee et al. 2017 [22]	Longitudinal	South Korea/37° N *	9839	OR = 3.1 (95% CI: 1.9–4.8)	- Low education - Longer working hours

OR = xx (yy−zz): Odds Ratio (Confidence Interval at 95% level); OWs: outdoor workers; prev: prevalence; pter: pterygium; * Seoul latitude.

**Table 3:** Synthesis of the studies on occupational solar UV radiation exposure and pterygium included in the systematic review of scientific literature published in the period 2008–2017 performed in World areas at high risk considering the mean annual UV index (UVI).

Study	Study Design	Place/Latitude	Sample Size (*n*)	Results of Association between Pterygium and Outdoor Work	Other Factors Associated with Pterygium
Rezvan et al. 2012 [23]	Cross-sectional	Iran/33° N	5190	Prev higher in OWs vs. indoor workers: 15% vs. 8%, and also vs. those working for a half of their time outdoor: 15% vs. 9.3% (*p* < 0.001)	- Prev = 9.4% (8.6–10.3), 2.9% (2.4–3.3) bilateral, increasing with age in women. - Male sex: prev 11% vs. 8% women - Low education: prev higher in illiterates vs. college education = 16.5% vs. 5.4% (*p* < 0.001)
Malekifar et al. 2017 [24]	Cross-Sectional Case-control	Iran/36° N	420	Higher frequency of OWs = *p* < 0.001 (univariate analysis) Farmers: OR = 3.84 (95% CI: 1.01–14.67) (*p* = 0.049)	- Higher frequency of welders = (*p* < 0.001, univariate analysis) - Inheritance, smoke, history of baking, age, severe blepharitis significantly associated in multivariate analysis = *p* < 0.001, *p* < 0.001, *p* = 0.002, *p* = 0.023, *p* = 0.002, respectively
Tano et al. 2013 [25]	Cross-sectional	Japan/37° N	2312	Prev slightly higher in OWs = 5.7% vs. 4.4%: OR = 1.47 (95% CI: 0.9–2.3) for any pter; OR = 1.95 (95% CI: 0.98–3.9) for bilateral pter (not significant)	- Age (10 years classes): OR = 1.36 (95% CI: 1.04–1.8) for any pter - OR = 1.8 (95% CI: 1.1–2.9) for bilateral
West et al. 2009 [26]	Cross-sectional	US/33° N	4774	OWs more likely males, with low economic income and low education: pter positively associated to all	- Male sex: OR = 2.67 (95% CI: 2.2–3.2) - Low education: <6 years OR = 2.8 (95% CI: 2.2–3.6); 6–11 vs. OR = 1.8 (95% CI: 1.4–2.2) - Low economic income: *p* < 0.001 - Bilateral cataract surgery (*p* = 0.004); diabetes (*p* = 0.03); smoke (*p* = 0.02) - Hypertension, alcohol use, any cataract not associated

OR = xx (yy−zz): Odds Ratio (Confidence Interval at 95% level); OWs: outdoor workers; prev: prevalence; pter: pterygium.

**Table 4:** Synthesis of the studies on occupational solar UV radiation exposure and pterygium included in the systematic review of scientific literature published in the period 2008–2017 performed in World areas at very high risk considering the mean annual UV index (UVI).

Study	Study Design	Place/Latitude	Sample Size (*n*)	Results of Association between Pterygium and Outdoor Work	Other Factors Associated with Pterygium
Sherwin et al. 2008 [27]	Cross-sectional	Australia/29° S	641	Spending more than 75% of the day outside: OR = 2.22 (95% CI: 1.20–4.09, *p* = 0.011) (Males who spent more than 75% of the day outside were more likely to be OWs)	- Male sex= prev 15% vs. 7.7% in females (*p* = 0.003) - Tanning skin phenotype OR = 2.17 (95% CI: 1.20–3.92) - Conjunctival UV auto-fluorescence (UVAF)/10 mmq: OR = 1.2 (95% CI: 1.2–1.3)
Zhong et al. 2012 [28]	Cross-sectional	South China/25° N	2133	OR = 1.51 (95% CI: 1.10–1.92)	- Age: OR ranged from 1.47 to 1.79 according to age groups - Female sex: OR = 1.42 (95% CI: 1.1–1.9) - Low education: OR = 1.3 (95% CI: 1.0–1.6) - BMI, hypertension, diabetes, smoking and alcohol use not associated
Li et al. 2015 [29]	Longitudinal	South China/25° N	2133	OR = 2.52 (95% CI: 1.27–4.95)	- 5 years pter incidence higher in women than in men (8.8% vs. 3.8%; *p* = 0.003)
Shiroma et al. 2009 [30]	Cross-sectional Case-control	South Japan/26° N	3762	OR = 1.82 (95% CI: 1.33–2.50) OWs were 77.8% in pter group vs. 59.0% in controls (*p* < 0.001). Farmers were the 31.8% of the pter group vs. the 22.4% of the controls (*p* < 0.001); fishermen were 4.2% vs. 2.4% (*p* = 0.003).	- Prev = 30.8% (13.1% bilateral), increasing with age OR = 1.02 (95% CI: 1.01–1.03), *p* < 0.001, male sex OR = 1.33 (95% CI: 1.03–1.63), *p* = 0.02 - Refractive error OR = 1.08 (95% CI: 1.03–1.13), *p* = 0.001 - Intraocular pressure OR = 0.96 (95% CI: 0.94–0.98), *p* = 0.002
Veena et al. 2013 [31]	Longitudinal	North India/26° N	200	Positive association with mean number of working hours outdoor = range 6–8/day	/
Chen et al. 2013 [32]	Cross-sectional	Taiwan/23° N	2197	OR = 1.47 (95% CI: 1.17–1.86)	- Age: prev = 25.2%, increased to 30.1% in 60–70 years group (*p* < 0.0001) - Male sex: OR = 1.31 (95% CI: 1.08–1.60) - Smoke: OR = 1.36 (95% CI: 1.02–1.83) - Live at seaside: OR = 1.6 (95% CI: 1.3–2.0)
Lin et al. 2016 [33]	Cross-sectional	Hawaii (U.S.)/21° N	169	History of outdoor occupation: *p* = 0.04	- Lifetime surfing hours (*p* < 0.0001) - Hawaiian residence (*p* = 0.009) - Hawaiian/Pacific Islander ethnicity (*p* = 0.002)

OR = xx (yy−zz): Odds Ratio (Confidence Interval at 95% level); OWs: outdoor workers; prev: prevalence; pter: pterygium.

**Table 5:** Synthesis of the studies on occupational solar UV radiation exposure and pterygium included in the systematic review of scientific literature published in the period 2008–2017 performed in World areas at extremely high risk considering the mean annual UV index (UVI).

Study	Study Design	Place/Latitude	Sample Size (*n*)	Results of Association between Pterygium and Outdoor Work	Other Factors Associated with Pterygium
Essuman et al. 2014 [34]	Longitudinal	Ghana/5° N	60	No significant association of pter recurrence with OW	- (overall frequency of pter recurrence = 37%)
Achigbu and Ezepue 2014 [35]	Cross-sectional	Nigeria/5–7° N	615	Length of employment as commercial riders: *p* = 0.009	- Lack of protective effect of regular use of sunglasses during work: *p* = 0.19
Anbesse et al. 2017 [36]	Cross-sectional	Ethiopia/12° N	390	OR = 3.8 (95% CI: 2.18–6.46)	- Regular use of sunglasses and hat: OR = 0.40 (95% CI: 0.2–0.78) - Age 41–60 years: OR = 2.20 (95% CI: 1.2–3.4) - Age 61–86 years OR = 7.97 (95% CI: 2.7–23.2) - Male sex: OR = 2.20 (95% CI: 1.28–3.82) - Traditional eye medications use: OR = 2.55 (95% CI: 1.04–5.9) - Inheritance: OR = 6.68 (95% CI: 2.53–17.6)
Nemesure et al. 2008 [37]	Longitudinal	Barbados/13° N	1888	OR = 1.51 (95% CI: 1.05–2.16, *p* < 0.001)	- Incidence not associated with aging - Darker skin: OR = 0.67 (095% CI: .46–0.97) - Glasses use: OR = 0.58 (95% CI: 0.4–0.8)
Coutts et al. 2011 [38]	Cross-sectional	Brazil/3° S	225	OWs = 70% of the sample, higher in rainforest inhabitants, where there is also higher pter prev. In subjects living along the riverside: OWs have the 93% of grade 3 pter and the 100% of most severe forms.	- Prev = 52% in urban contest, 62.5–75% in rural places
Asokan et al. 2012 [39]	Cross-sectional	South India/13° N	7774	Type of work not associated Rural residence, high lifetime UV exposure (*p* < 0.0001)	- No use of glasses: OR = 1.4 (95% CI: 1.1–1.8) - Smoking, use of alcohol, diabetes, hypertension not associated
Cherian et al. 2013 [40]	Cross-sectional	South India/12° N	331	Prev = 13% in salt workers exposed to reflected UV rays	- Prev higher in males
Marmamula et al. 2013 [41]	Cross-sectional	South India/16–19° N	10,293	OR = 1.8 (95% CI: 1.5–2.2, *p* < 0.001)	- Prevalence increases with age - Living in rural areas: OR = 1.8, (95% CI: 10.9–12.6) - Higher education level: OR = 0.6 (95% CI: 0.5–0.7; *p* < 0.001)
Salagar et al. 2013 [42]	Longitudinal	South India/17° N	100	OWs were the 80% of the patients treated, and those with most complicated forms: *X*_2_ = 7.7, *p* < 0.001	
Artornsombudh et al. 2013 [43]	Cross-sectional	Thailand/13° N	482	53.7% of the sample were farmers, 18.7% laborers, 4.6% merchants, 1% truck drivers, 22% indoor workers.	- Prev of conjunctival epithelial neoplasia = 1.8% in pter patients
Cajucom et al. 2010 [44]	Cross-sectional	Singapore/1° N	3280	OR = 2.2 (95% CI: 1.1–4.5, *p* = 0.03) for severe pterygium (grade 3).	- Age: OR = 1.3 (95% CI: 1.1–1.4) - Male sex: OR = 1.9 (95% CI: 1.5–2.6) - Hypertension: OR = 1.6 (95% CI: 1.2–2.1)
Ang et al. 2012 [45]	Cross-sectional	Singapore/1° N	8906	OR = 2.1 (95% CI: 1.1–4.0; *p* = 0.02) for severe pter	- Ethnicity (*p* < 0.001): Malays = prev 15.5%; Chinese = 7.0%; Indians = 7.0% - Age (*p* < 0.001), male sex (*p* < 0.001), low education level (*p* < 0.001)

OR = xx (yy−zz): Odds Ratio (Confidence Interval at 95% level); OWs: outdoor workers; prev: prevalence; pter: pterygium.

**Table 6:** Mean pterygium prevalence in the studies reviewed according to the UV risk areas: evaluation of the differences.

UVI Risk Area	Study	Pterygium Prevalence (%)	Total Number of Subjects Included in the Prevalence Estimates per Area	Average Pterygium Prevalence per Area (%)	
				Weighted for the Number of Subjects	Crude (Range) According to the Number of Studies
UVI ≤ 5 (MODERATE)	Viso et al. 2011 [17]	5.9	41,497	5.5	4.8 (2.5–7.1)
	Zhao et al. 2013 [18]	2.5			
	Li et al. 2014 [19]	2.5			
	Lim et al. 2015 [20]	6.2			
	Nam et al. 2016 [21]	7.1			
UVI ≥ 6 (HIGH + VERY HIGH + ESTREMELY HIGH)	Rezvan et al. 2012 [23]	9.4	55,180	14.9	19.3 (4.4–38.7)
	Tano et al. 2013 [25]	4.4			
	West et al. 2009 [26]	16.2			
	Sherwin et al. 2008 [27]	10.9			
	Zhong et al. 2012 [28]	39			
	Shiroma et al. 2009 [30]	30.8			
	Chen et al. 2013 [32]	25.2			
	Lin et al. 2016 [33]	11.9			
	Achigbu & Ezepue 2014 [35]	13.3			
	Anbesse et al., 2017 [36]	38.7			
	Nemesure et al. 2008 [37]	19.3			
	Coutts et al. 2011 [38]	52			
	Asokan et al. 2012 [39]	9.5			
	Cherian et al. 2013 [40]	13			
	Marmamula et al. 2013 [41]	11.7			
	Cajucom et al. 2010 [44]	12.3			
	Ang et al. 2012 [45]	10.1			

**Table 7:** Mean Odds Ratios for likelihood of Outdoor Workers to have pterygium in the studies reviewed according to the UV risk areas: evaluation of the differences.

UVI Risk Area	Study	Odds Ratio (OR) for Outdoor Work (OW)	Mean OR in the Risk Area
UVI ≤ 5 (MODERATE)	Viso et al. 2011 [17]	2.3	2.2
	Zhao et al. 2013 [18]	2.3	
	Li et al. 2014 [19]	1.8	
	Lim et al. 2015 [20]	1.5	
	Lee et al. 2017 [22]	3.1	
UVI ≥ 6 (HIGH + VERY HIGH + ESTREMELY HIGH)	Malekifar et al. 2017 [24]	3.8	2.2
	Tano et al. 2013 [25]	1.5	
	Sherwin et al. 2008 [27]	2.2	
	Zhong et al. 2012 [28]	1.5	
	Li et al. 2015 [29]	2.5	
	Shiroma et al. 2009 [30]	1.8	
	Chen et al. 2013 [32]	1.4	
	Anbesse et al. 2017 [36]	3.8	
	Nemesure et al. 2008 [37]	1.5	
	Marmamula et al. 2013 [41]	1.8	
	Cajucom et al. 2010 [44]	2.2	
	Ang et al. 2012 [45]	2.1	
